# Scarlatinal Dropsy, with Suppression of Urine and Convulsions

**Published:** 1873-03-15

**Authors:** E. S. Cleveland

**Affiliations:** Lawrence, Mich.


### SCARLATINAL DROPSY, WITH SUPPRESSION OF URINE AND CONVULSIONS.

BY E. S. CLEVELAND, LAWRENCE, MICH.

In the number of MEDICAL EXAMINER for Feb. 1st, I noticed a clinical report of two cases of scarlatinal dropsy, with suppression of urine and convulsions.

I wish to report two cases of the same nature which fell under my care, to illustrate the value of the gelsemium sempervirens in their treatment.

Gelsemium is one of the most efficient and powerful anti-spasmodics in the materia medica. In addition, it relieves irritation and determination to the kidneys; and, in larger doses, causes a copious secretion of urine. It is an excellent remedy in some cases of urinary retention, and is reported to have been used successfully in cases of tight strictures, enabling the operator to pass a bougie through a previously "impermeable" stricture.

CASE I.—I was called, on the night of May 4th, 1870, to an adjoining township, to see Miss Liula Heath, aged thirteen.

Three weeks previously had had a very light attack of scarlatina simplex; convalescing without having had a physician. For several days her mother had noticed slight bloating about the eyes, and that the urinary secretion was less than it should be, and of a smoky-brown color. I reached the house toward midnight, and learned that no urine had been passed since morning, and that she had been in convulsions since dark. There was nausea and constipation. Having been misinformed as to the nature of her disease, I had carelessly started, expecting that I had all the medicine I needed in my vest pockets, —an exploration of which showed that gelsemium was my only weapon in this case (I was six miles from town). I administered fifteen drops of the fluid extract, and ordered hot cloths to be kept constantly over the loins, to be changed before they became cold. Continued the gelsemium in doses of ten drops every hour. I stayed long enough to see that the remedy was exerting its effect as an anti-spasmodic, the convulsions at once becoming lighter, and the intervals longer.

May 5th—Noon.—Has had two convulsions since my last visit; passed a small amount of urine, dark brown in color, at five this morning; nausea diminished. Gelsemium has not affected her eyelids. Continued the treatment, only, in addition, gave jalap and bi-tart. potassa, to move the bowels.

May 6th.—Face and limbs much bloated; passed a small amount of urine twice yesterday, of same appearance as that I saw in the morning; had two slight spasms during the night; bowels had moved twice; passed three or four ounces of urine while I remained in the house; eyelids heavy. Lengthened the interval between the doses of gelsemium to four hours, giving spts. nitre and acetate of potash. The next day omitted gelsemium, and added tinct. iron. I cannot find any note of amount of albumen in this case, though I tested the urine several times. She convalesced in ten days. This was my first case of the kind, and, in fact my introduction to scarlet fever and sequels —though in the succeeding two years we had a very malignant epidemic of the disease.

CASE II.—Dec. 4, 1871, was called, in haste, to see Miss Bertha Combs, aged ten. Two weeks ago, had a slight attack of scarlatina, which neither confined her to her bed, nor to the house. For several days she has been dropsical, and urine scanty, for which I sent a prescription two or three days since, though I did not see the case. Found, on my arrival, at 10 P.M., that she had passed no urine since previous day. In morning had vomited, and had been sick at stomach more or less since. Dropsy had increased very much, and she was drowsy all the forenoon. At noon was seized with convulsions, which had continued increasing in intensity ever since. She was in the midst of one when I entered, which lasted, her mother said, twenty minutes. The length of the interval was generally ten minutes, in which she seemed almost comatose; could scarcely be aroused, nor did she understand anything which was said to her.

As soon as she could swallow, I gave her twenty drops of fluid extract gelsemium. Scarified and cupped her on the loins to three ounces, and applied cloths wrung out in water as hot as could be borne, ordering them changed every thirty minutes. At the end of an hour gave twenty drops more. She remained free from convulsions for an hour and twenty minutes from the first one I saw, when she had a slight one, which was the last. She still remained comatose. Gave twenty grs. comp. powder of jalap, and repeated it in three hours; continued the gelsemium in two drop doses every hour, and the hot cloths.

At 4 A.M., Dec. 5th, bowels moved thoroughly twice. The last time she also passed a small amount of bloody urine. 5 A.M., passed a couple of ounces of urine, of smoky-red color. 6 A.M., roused up and recognized me; asked when I came, and how long I had been there, and spoke to her father and mother.

Prescribed acetate of potash, in two-grain doses, to be taken in an infusion of hair-cap moss every three hours, and went home. 3 P.M., has passed urine freely several times; tested for albumen; the coagulum filled half the test-tube, which was less than I expected. Ordered the acetate of potash, to be continued to the amount of one drachm a day, in the infusion of moss, and a teaspoonful of the following, three times daily:

℞.—Tinct. ferri chlor., ℥ ii.

Glycerine, } aa., ℥ ii.

Syrup, }

She convalesced rapidly.

Six children in this family had the scarlet fever, and, in three of them, it was followed by dropsy.

In the first case, that of Jeff, aged twelve, the dropsy exceeded anything I have ever seen; and the urine discharged did not exceed four ounces a day. He is a very witty, active lad, and the only effect visible from this condition of things was a disinclination to move away from the stove, "because he couldn't bend his legs." He was, if possible, sharper than ever.

With one exception, I have never seen a quicker effect from gelsemium than in the second case reported; and, though it was given in full doses, it did not affect her eyelids at all.

The physicians in this vicinity were very much pleased with the action of the hair-cap moss (*polytrichum juniperium*). Increasing the watery element of the urine, and, being unirritating, it was an excellent vehicle for the saline.

The epidemic began in the summer of 1871 (a few cases having been seen during the previous year), in a very malignant form, most of the first cases dying in a few hours from the attack, one family losing three children in thirty-six hours.

It furnished an unusual number of severe cases, and almost every mild case which came under my notice exhibited more or less anasarca as a sequel. Affections of the glands, as a sequel, was rare in any class.

