# Correction

**Published:** 1873-04-01

**Authors:** 


CORRECTION.—In the article of E. S. Cleveland, in our March 15 number, acetate of potash, two grains, should read twelve grains; and tinct. ferri, half an ounce, should read two ounces.

